# Can patient involvement improve patient safety? A cluster randomised control trial of the Patient Reporting and Action for a Safe Environment (PRASE) intervention

**DOI:** 10.1136/bmjqs-2016-005570

**Published:** 2017-02-03

**Authors:** Rebecca Lawton, Jane Kathryn O'Hara, Laura Sheard, Gerry Armitage, Kim Cocks, Hannah Buckley, Belen Corbacho, Caroline Reynolds, Claire Marsh, Sally Moore, Ian Watt, John Wright

**Affiliations:** 1 Institute of Psychological Sciences, University of Leeds, Leeds, UK; 2 Department of Quality and Safety Research, Bradford Institute for Health Research, Bradford, UK; 3 Leeds Institute of Medical Education, University of Leeds, Leeds, Leeds, UK; 4 Quality and Safety Research Group, Bradford Institute for Health Research, Bradford, Bradford, UK; 5 School of Health, University of Bradford, Bradford, Bradford, UK; 6 York Trials Unit, Department of Health Sciences, University of York, York, UK; 7 York Trials Unit, University of York, York, UK; 8 Department of Quality and Safety, Bradford Institute for Health Research, Bradford, UK; 9 Department of Health Sciences, The University of York, York, North Yorkshire, UK; 10 Department of Epidemiology and Public Health, Royal Infirmary Bradford, Bradford, UK

**Keywords:** Patient safety, Randomised controlled trial, Patient-centred care, Cluster trials, Healthcare quality improvement

## Abstract

**Objective:**

To evaluate the efficacy of the Patient Reporting and Action for a Safe Environment intervention.

**Design:**

A multicentre cluster randomised controlled trial.

**Setting:**

Clusters were 33 hospital wards within five hospitals in the UK.

**Participants:**

All patients able to give informed consent were eligible to take part. Wards were allocated to the intervention or control condition.

**Intervention:**

The ward-level intervention comprised two tools: (1) a questionnaire that asked patients about factors contributing to safety (patient measure of safety (PMOS)) and (2) a proforma for patients to report both safety concerns and positive experiences (patient incident reporting tool). Feedback was considered in multidisciplinary action planning meetings.

**Measurements:**

Primary outcomes were routinely collected ward-level harm-free care (HFC) scores and patient-level feedback on safety (PMOS).

**Results:**

Intervention uptake and retention of wards was 100% and patient participation was high (86%). We found no significant effect of the intervention on any outcomes at 6 or 12 months. However, for new harms (ie, those for which the wards were directly accountable) intervention wards did show greater, though non-significant, improvement compared with control wards. Analyses also indicated that improvements were largest for wards that showed the greatest compliance with the intervention.

**Limitations:**

Adherence to the intervention, particularly the implementation of action plans, was poor. Patient safety outcomes may represent too blunt a measure.

**Conclusions:**

Patients are willing to provide feedback about the safety of their care. However, we were unable to demonstrate any overall effect of this intervention on either measure of patient safety and therefore cannot recommend this intervention for wider uptake. Findings indicate promise for increasing HFC where wards implement ≥75% of the intervention components.

**Trial registration number:**

ISRCTN07689702; pre-results.

## Introduction

Rates of adverse events during hospitalisation have been estimated at between 3% and 16% globally and, despite increasing attention, have demonstrated very little improvement over the last 10 years.^1^ The role that patients could play in promoting safety and reducing adverse events is now an international policy priority. For example, the WHO's World Alliance for Patient Safety cites mobilisation and empowerment of patients as one of six action areas that will be taken forward in its ‘Patients for Patient Safety’ programme.^2^ Despite international emphasis and repeated calls for greater patient involvement,^3^ Wachter referred to the lack of progress in this area as a ‘troubling gap’, when rating the achievements of healthcare in promoting safety.^4^ Perhaps even more troubling is the dearth of research evidence on how best to involve patients and whether such involvement leads to improvements in safety. The evidence that does exist indicates that patients are willing and able to participate in error prevention strategies^5^ that have the potential to improve safety.^6–9^ However, there can be a reluctance to challenge staff and provide negative feedback that might directly impact on the quality of their care,^10^ ^11^ and there is a risk of shifting responsibility for safety onto patients.^12^ Patient involvement comes in a variety of forms, from patient education about risk through to involvement in monitoring the safety practices of healthcare professionals. The potential for such interventions to improve communication and patient experience as well as save money and improve outcomes is great,^13^ and early findings suggest that such outcomes are feasible.^14^ However, research in this field is lacking, with systematic reviews concluding that there is limited and poor quality evidence that patient involvement has any benefits for patient safety.^15–17^

Previous work^18–20^ has described the development of tools, based on Reason's model of organisational safety,^13^ that allow patients to provide feedback on the safety of their care environment to inform local and organisational changes. One tool, patient measure of safety (PMOS), asks patients to report on those factors that have been identified in the literature^21^ and by patients^18^ as contributing to patient safety. These include communication and teamwork, roles and responsibility, and ward environment. The second tool, patient incident reporting tool (PIRT)^22^ asks patients to report on patient safety incidents they have experienced or witnessed as well as any positive experiences. Together, information from these two tools is assimilated and feedback provided to ward teams, who are asked to convene as a multidisciplinary group to consider the feedback and to make and implement action plans based on this information. The feasibility of this intervention known as Patient Reporting and Action for a Safe Environment (PRASE) has been reported elsewhere.^23^ Here, we evaluate the costs and benefits of the PRASE intervention and its effect on measures of patient safety when implemented on hospital wards. This study represents the first randomised control trial (RCT) of the effectiveness of a patient feedback intervention to improve patient safety.

## Methods

This evaluation employed a multicentre, cluster, waitlist, RCT design to assess the efficacy of the PRASE intervention in achieving patient safety improvements over a 12-month period. The published protocol for the trial is available elsewhere.^20^

### Participants

This study was conducted in 33 hospital wards, across five hospitals (three National Health Service (NHS) Trusts) in the UK. At the small district hospital, all adult non-intensive wards were recruited (N=9). At the medium-sized teaching hospital Trust, the chief nurse recruited 10 adult wards to the study. Within the large Trust, wards (N=14) were asked to volunteer to take part. An average of 25 patients meeting the following eligibility criteria within each ward were recruited at three different time points: ≥aged 16, able to give informed consent and minimum period of 4 hours on the ward before questionnaire administered. Patients were excluded if they were too ill or distressed to take part and had already been in the study within the previous month or were non-English or non-Mirpuri-speaking patients.

### Intervention

The PRASE intervention uses two theoretically informed and validated tools to collect patient feedback about the safety of care as a means of achieving patient-centred service improvement (see logic model in online supplementary appendix 1). The first tool, PMOS, is a 44-item questionnaire asking patients to report on a range of upstream factors that contribute to safety, for example, communication and teamwork, physical environment, staff roles and responsibilities.^18^ ^19^ The second, PIRT, asks patients to report on any safety concerns they have experienced during their inpatient stay and provide detail about what happened, why they felt it happened, what could be done to prevent it happening again, and the severity and preventability of the concern from their (non-clinical) perspective.

10.1136/bmjqs-2016-005570.supp1 supplementary appendix

This patient feedback is then collated and presented to each ward as part of a multidisciplinary meeting during which ward staff are supported to agree a set of ward-specific actions to address areas of patient concern. The philosophy of this intervention is that it is an iterative process with a cycle of measurement, feedback and change lasting for a period of 6 months. Ward staff engaged in two cycles during the 12-month intervention period. Following the feasibility study,^22^ changes were made to the intervention to support effective implementation. The research team facilitated action planning meetings using an action planning proforma to capture the ward improvement plans. We also launched the project in Trusts at a start-up meeting and hosted a meeting after the first set of action planning meetings had been completed and another at the end of the project. Senior managers were invited to attend these meetings and agreed to support the wards in delivering change.

Researchers were equally visible on both the control and intervention wards, as data were collected for the purposes of feedback as part of the intervention and outcome measurement. Control wards received their PRASE feedback in a single report at the end of the 12 months follow-up period accompanied by a 1-hour training session on how to interpret and use this feedback.

### Intervention fidelity

Assessment of fidelity followed a previously published framework^24^ and was informed by the intervention's logic model. We conducted a detailed process evaluation to understand how the intervention was received and used by each of the 17 intervention wards (submitted for publication). Adherence was assessed in relation to eight components of the intervention listed below; each was rated independently by three members of the research team and, through discussion, consensus was achieved. These scores were based on data collected by senior researchers (LS, JKO and CM) who (1) observed and recorded attendance at all meetings, (2) obtained copies of all action plans developed by ward teams and (3) conducted a telephone follow-up with the nominated PRASE lead for each ward and recorded the extent to which each action plan was reported to have been implemented. Box 1 shows the scoring of the fidelity of implementation of each of the eight intervention components.

Box 1 **Measurement of Patient Reporting and Action for a Safe Environment implementation fidelity**

- Attendance of at least one ward representative at orientation meeting (0=no; 3=yes)
- Multidisciplinary team present at the phase I action planning meeting (APM) (0=no APM; 1 = one staff group represented; 2 = at least two staff groups represented and 3 = more than two staff groups represented)
- Creation of action plans (APs) in phase I (0= no APs; 1= limited APs including mainly quick fixes; 2 = considered APs reflecting issues identified with potential for short-term impact and 3 = as 2, but potential for longer-term solutions)
- The extent to which APs were implemented in phase I (0= no implementation; 1= at least one AP partially implemented; 2 = most APs implemented and 3 = all APs implemented)
- Multidisciplinary team present at the phase II APM (as above)
- Creation of APs in phase II (as above)
- Implementation of APs in phase II (as above)
- Attendance of at least one ward representative at midpoint meeting (as orientation meeting)

It was decided a priori that the values of 0 and 1 on each of the components would be classed as non-adherence while scores of 2 or 3 would imply adherence. Thus, overall fidelity scores ranged from 0 (non-adherence with all components) to 8 (adherence to all eight components) for each ward.

### Outcomes

Each hospital attended a start-up meeting at which the study was introduced. Baseline data collection then proceeded on all wards in participating hospitals after which the wards were then randomised to the two groups. Follow-up data collection occurred at 6 and 12 months post randomisation on all wards.

### Patient safety thermometer

The patient safety thermometer (PST) is a ward-level routinely collected compulsory measure of patient harm that was introduced in the NHS in 2012 shortly prior to the start of this trial. Information is collected on new and existing cases of four types of possible ‘harm’ (pressure ulcers, venous thromboembolism, catheter- associated urinary tract infections and falls) from all patients on a ward on a single day each month. The key outcome for analysis was harm-free care (HFC) at the ward level that can range between 0% and 100%; higher scores indicate greater HFC (so are positive). A baseline was calculated using the average of the 3 months prior to randomisation; for the 6-month and 12-month time points, the month during the collection of patient feedback and the preceding and following months were averaged. Following experience with the measure during the trial, a summary from the PST focusing on new harms only was added as a key outcome. This excluded historical harms occurring prior to admission of the patient to the specific ward and was therefore deemed a more sensitive measure of the efficacy of the intervention.

### Patient measure of safety

The 44-item PMOS questionnaire was completed by patients as part of the intervention and was also used as an outcome measure. Patients were given the choice of completing the questionnaire themselves via a tablet computer or providing responses verbally, which the researcher then input. Forty-three items were scored on Likert scales from 1 to 5; one additional item also required a qualitative response. An overall PMOS score was calculated for those responding to at least 80% of items by averaging over item scores; overall scores were means that ranged from 1 to 5, with high scores indicating more positive response.

### Secondary outcomes

Three secondary outcome measures were also measured. First, participants were asked three Commissioning for Quality and Innovation (CQUIN) questions, which are measured within the NHS Inpatient Survey^25^ (example question: “Were you involved as much as you wanted to be in decisions about your care and treatment?”). Second, participants were asked to complete the ‘NHS Friends and Family Test’ question:^26^ “How likely are you to recommend this ward to friends and family if they needed similar care or treatment?”. Both of these measures are routinely collected within the NHS as patient-reported indicators of quality of care. Third, we measured staff perceptions of safety culture using the four outcome questions from the Hospital Survey of Patient Safety.^27^ For further details of secondary outcome measures collected, see the published protocol.^20^

### Outcome data collection

PMOS data were collected by research nurses during three periods over 12 months (at baseline, and 6 and 12 months post randomisation). Informed consent was taken prior to data collection. Research nurses visited each ward on a daily basis until a minimum of 20 patients were recruited. Routinely available PST data were extracted via the Health and Social Care Information Centre website.^28^ Data for the purposes of the assessment of fidelity (see above) were collected by LS, CM and JKO and were coded by LS, GA and RL.

### Sample size

The study was powered to detect a small-to-medium difference (effect size (ES) =0.3) between the allocated groups with respect to the PMOS score. A small-to-medium ES seemed a reasonable assumption as each ward would be focusing on developing and implementing their own action plans, tailored using their initial feedback. The intervention was therefore specific to individual wards and was not expected to impact on all areas measured by the PMOS. Any improvement on the PMOS overall score, which summates across various domains, was therefore likely to be small. In order to achieve 80% power (with alpha=0.05) with an average cluster size of 25 patients and assumed intraclass correlation coefficient (ICC) of 0.05, 32 wards were required. This estimate of ICC seemed reasonable for a trial in secondary care with a patient-reported outcome.^29^

The PST was newly introduced shortly prior to the start of the trial, and the way in which the data would be formatted (eg, individual data, ward level) for public availability was unclear at the time.

### Randomisation

Wards were randomly assigned to the intervention or control group on an equal basis by York Trials Unit. Minimisation was used to balance groups with respect to ward type (medical or surgical), age (low, middle or high based on tertiles), male/female/mixed sex wards and ward size (low, middle or high based on tertiles). It was not possible to blind wards to group allocation, but research nurses who collected data were blinded.

### Statistical methods

Analysis was conducted in Stata V.13 using the principles of ‘intention-to-treat’, meaning that wards (and associated patients) were analysed according to their randomised trial arm regardless of intervention implementation or fidelity. Minimisation factors and clustering were accounted for where required; all ES s are Cohen's d.^30^ Statistical significance was assessed at the two-sided 5% level. Analyses for PST and Hospital Survey of Patient Safety were conducted at the ward level; all other outcomes were available and analysed at the patient level.

The primary analysis on the PST outcome used a ward-level linear regression model with weighting to assess differences between the allocated groups in the percentage of HFC at 12 months. Adjustment was made for baseline HFC and minimisation factors. Two sensitivity analyses were preplanned, an unadjusted analysis and the primary model adjusted for ward-level characteristics. Two further post hoc sensitivity analyses were carried out, one repeated the analysis including one ward with partially missing baseline data and the second analysed the data using a repeated measures logistic regression model with random effects for ward. The primary analysis was repeated considering new HFC as was the repeated measures model. A linear mixed model with random effects to account for clustering at the ward level and fixed effects for baseline ward average and minimisation factors were used for analysis of the 12-month PMOS data. Cronbach's alpha was used to assess the internal reliability (alpha >0.7) of the PMOS. Complier average causal effect (CACE) analysis was applied to account for non-adherence.

Secondary analyses repeated the primary models at 6 months. Similar regression models were used to analyse the CQUIN and Hospital Survey of Patient Safety outcomes at the patient and ward level, respectively.

For the cost-consequences analysis, we estimated resource use related to (1) collection of patient data; (2) action planning meetings; (3) management of the PRASE intervention via start-up, midpoint and closing meetings with senior managers and all teams in a hospital; and (4) actions. Resource use was estimated in terms of the mean value per ward. Unit costs were retrieved from Personal Social Services Research Unit Costs of Health and Social Care 2013.^31^

## Results

### Participant flow and recruitment

**Figure 1:**
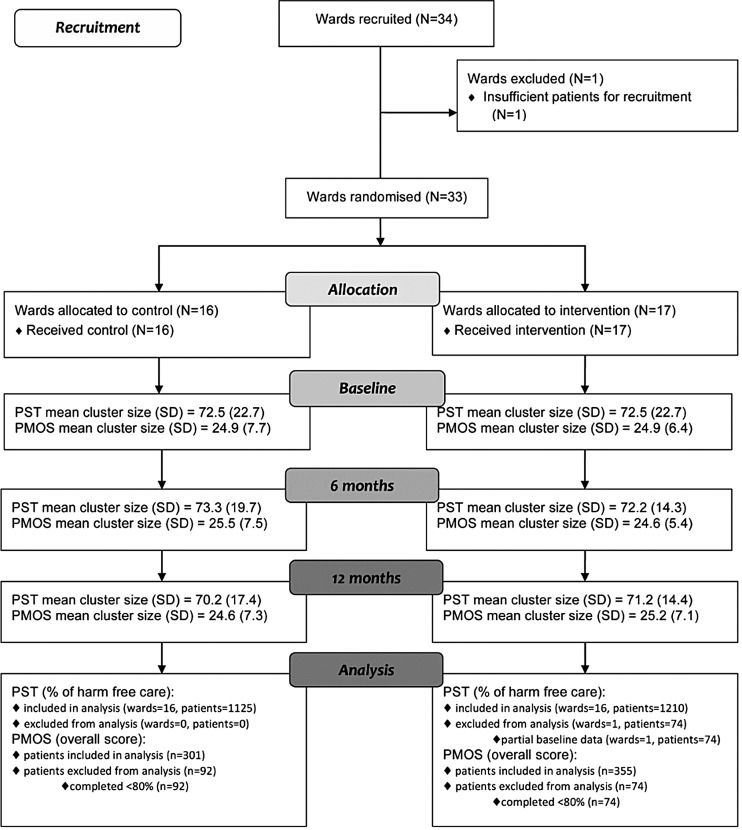
Consolidated Standards of Reporting Trials diagram relating to patient safety thermometer (PST) and patient measure of safety (PMOS) data.

Thirty-four wards were recruited, two extra than required to allow for drop out. One ward was excluded prior to randomisation due to insufficient number of patients available (figure 1). Thirty-three wards were randomised (16 allocated to control, 17 to intervention). All wards were retained throughout the trial. Participant data collection periods ran between May and July 2013, January and April 2014 and June and September 2014.

The percentage of patients approached who provided feedback was 89%, 88% and 82% for each time point, respectively (86% overall). For PST, average cluster sizes were large as 3 months worth of data was used at each time point in order to capture the data over the same time period as the PMOS; average PMOS cluster sizes were 25 patients as planned.

### Fidelity of the intervention

**Figure 2:**
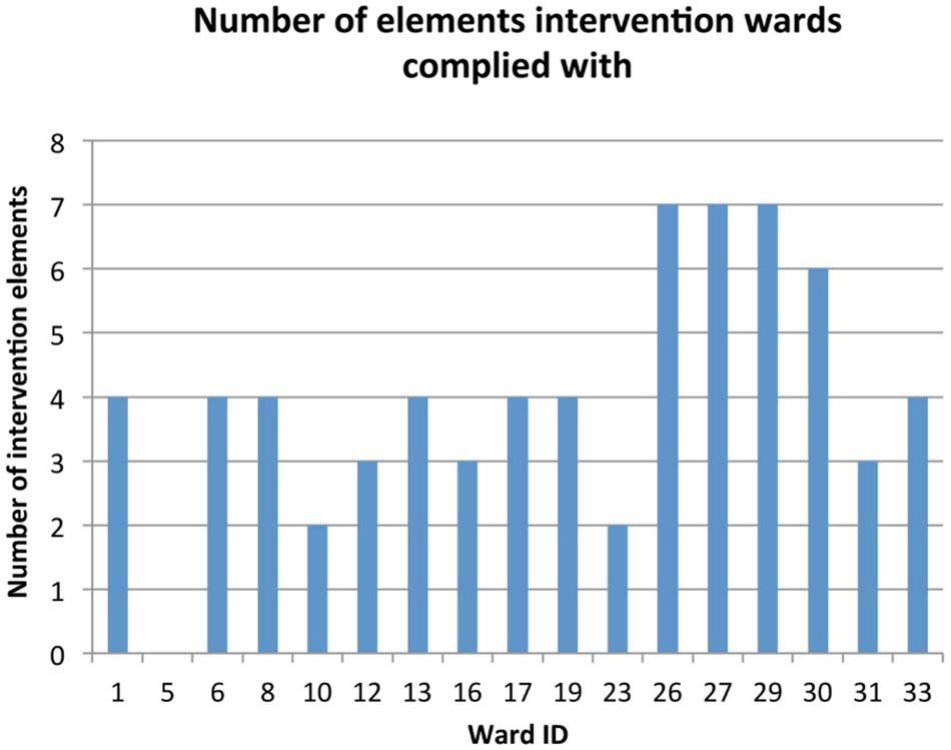
Compliance with elements of the intervention for each of the 33 wards at 12 months.

At 12 months, 11 of the 17 (64.7%) intervention wards complied with at least 50% of the intervention components; a total of four intervention wards (23.5%) complied with at least 75%. These data are shown in figure 2. One ward barely engaged with the intervention in either cycle, did not make an action plan and in the second cycle only the ward manager met to consider feedback. This ward^5^ scored zero on implementation fidelity. Where adherence was low, this was because wards struggled to pull a multidisciplinary team together to develop long-term solutions to problems and/or to implement action plans.

### Baseline data

Summary statistics for the minimisation factors are reported in table 1; all proportions were similar between allocated groups.

**Table 1:** Baseline ward-level characteristics by allocation group as minimised

	Control	Intervention
N	16	17
Average age*		
Low tertile, n (%)	5 (31.3)	4 (23.5)
Middle tertile, n (%)	4 (25.0)	5 (29.4)
High tertile, n (%)	7 (43.8)	8 (47.1)
Ward gender		
Female, n (%)	1 (6.3)	4 (23.5)
Male, n (%)	2 (12.5)	3 (17.7)
Mixed, n (%)	13 (81.3)	10 (58.8)
Ward type		
Medical, n (%)	7 (43.8)	8 (47.1)
Surgical, n (%)	9 (56.3)	9 (52.9)
Ward size†		
Low tertile, n (%)	4 (25.0)	6 (35.3)
Middle tertile, n (%)	3 (18.8)	4 (23.5)
High tertile, n (%)	9 (56.3)	7 (41.2)

*Low tertile = <59 years, middle tertile=59–64 years, high tertile=65+ years.

†Low tertile = <24 patients, middle tertile=24–28 patients, high tertile=28+ patients.

Patient-level summary statistics are presented in online supplementary appendix 2. Balance was observed on all variables (patient age, gender and ethnicity) at each time point between allocated groups.

10.1136/bmjqs-2016-005570.supp2 supplementary appendix

### Raw summary scores

Table 2 shows unadjusted summary statistics. At each time point, both the mean percentage HFC and percentage new HFC were high (>90). A ceiling effect was not observed in the PMOS scores with only three patients at the highest score of 5. Both PMOS and PST scores were similar between allocated groups at each time point. Routinely collected PST data were not available for one control ward at baseline.

**Table 2:** Raw summary statistics for primary outcomes by allocated group at each time point

		Patient measure of safety		Patient safety thermometer				
		Overall score		Percentage harm-free care			Percentage new harm-free care	
Timepoint	Group	Patients (n, %)	Mean* (SD)	Wards (N)	Mean† (SD)	(Min, Max)	Mean (SD)	(Min, Max)
Baseline		352 (88.2)	3.9 (0.4)	16‡	92.4 (5.0)	(82.4, 100)	96.5 (3.7)	(88.6, 100)
	Intervention	340 (80.4)	3.9 (0.4)	16	91.4 (7.3)	(72.0, 100)	97.0 (2.9)	(88.7, 100)
6 months	Control	382 (93.6)	3.9 (0.4)	16	94.1 (6.3)	(73.9, 100)	96.2 (5.5)	(79.7, 100)
	Intervention	385 (91.9)	3.9 (0.4)	17	94.0 (3.9)	(87.2, 100)	97.4 (2.8)	(90.8, 100)
12 months	Control	301 (76.6)	4.0 (0.4)	16	92.5 (3.5)	(85.9, 97.4)	95.8 (3.4)	(86.8, 100)
	Intervention	355 (82.8)	4.0 (0.4)	17	92.4 (6.7)	(73.1, 100)	97.7 (2.9)	(89.0, 100)

*Mean and SD based on patient-level data.

†Mean and SD based on ward-level data.

‡Routinely collected patient safety thermometer data unavailable for one control ward at baseline for unknown reasons.

### Primary analyses

**Figure 3:**
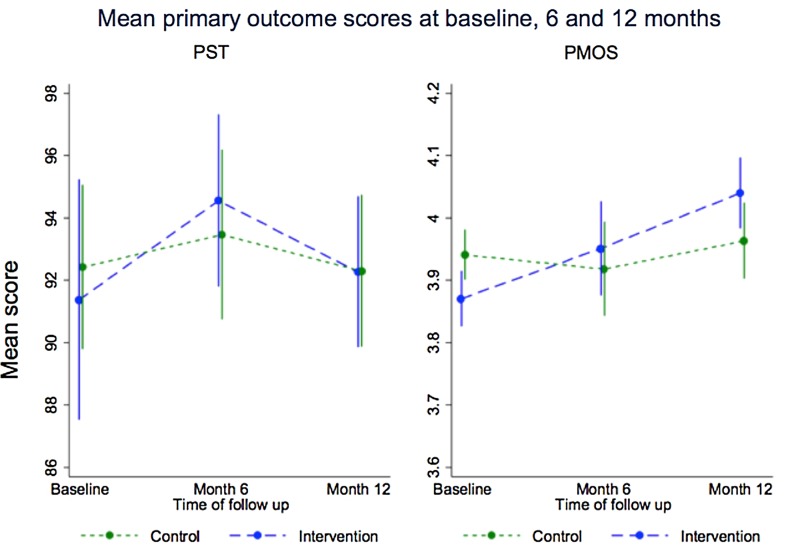
Mean primary outcome scores at baseline, 6 and 12 months. PMOS, patient measure of safety; PST, patient safety thermometer.

The reliability of the PMOS questionnaire was high at each time point (alpha>0.9). A linear mixed model, conducted on 656 individuals who completed ≥80% of the questionnaire items, showed a non-significant difference of 0.08 (p=0.09, 95% CI −0.01 to 0.17, ES =0.20) between the allocated groups in overall PMOS score at 12 months (table 3 and figure 3). The marginal mean was lower in the control group at 3.96 (95% CI 3.90 to 4.02) than in the intervention group (4.04, 95% CI 3.98 to 4.10). An ICC of 0.03 was observed; the lower-than-expected ICC counteracts the loss of power caused by exclusions due to incomplete questionnaires. Sensitivity analyses produced consistent results (table 3). Effects of the intervention were greater though still non-significant when compliance with the intervention was accounted for.

**Table 3:** Summary of 12-month primary outcome results

	Difference (95% CI)	p Value
Patient safety thermometer		
Primary analysis	−0.03 (−3.59 to 3.53)	0.99
No adjustment for minimisation factors	≈0.00 (−3.22 to 3.22)	1.00
With adjustment for ward characteristics	−1.03 (−4.63 to 2.57)	0.56
Inclusion of previously excluded ward	0.06 (−3.30 to 3.42)	0.97
Repeated measure logistic regression model	−0.05 (−0.53 to 0.44)*	0.84
CACE (50% compliance cut-off)	−0.06 (−5.61 to 5.50)	0.98
CACE (75% compliance cut-off)	−0.12 (−11.70 to 11.46)	0.98
New harm	1.60 (−0.62 to 3.83)	0.15
Repeated measure logistic regression model	−0.49 (−1.19, 0.22)*	0.17
New harm CACE (50% compliance cut-off)	2.42 (−1.38 to 6.22)	0.19
New harm CACE (75% compliance cut-off)	5.38 (−3.89 to 14.64)	0.24
Patient measure of safety		
Primary analysis	0.08 (−0.01, 0.17)	0.09
With adjustment for ward characteristics	0.05 (−0.04, 0.14)	0.27
With adjustment for method of completion	0.08 (−0.01, 0.16)	0.08
CACE analysis (50% compliance cut-off)	0.15 (−0.05, 0.36)	0.13
CACE analysis (75% compliance cut-off)	0.43 (−0.28, 1.14)	0.23

*Results on the logit scale.

CACE, complier average causal effect.

There was no evidence of a difference in HFC at 12 months between the allocation groups (p=0.99) in a ward-level analysis, with a non-significant decrease of 0.03% for intervention wards compared with control wards (95% CI −3.59 to 3.53, ES=−0.01). Adjusted mean HFC was 92.30% and 92.26% for control and intervention wards, respectively. Sensitivity analyses (table 3) produced consistent results.

### Harm-free care (excluding old harms)

When the percentage HFC score excluded old harms that occurred prior to the patient being on the participating ward, there was again no evidence of a significant difference between the allocated groups at 12 months (p=0.15) in a ward-level analysis; however, a larger increase of 1.60% (95% CI −0.62 to 3.83, ES=0.51) was observed for intervention over control wards. Results from the repeated measures logistic regression model conducted on individual-level data were consistent. CACE analysis showed greater improvements in HFC excluding old harms of 2.42% (50% compliance) and 5.38% (75% compliance) for the intervention group compared with the control when intervention fidelity was high.

### Secondary analyses

Regression models on PST and PMOS 6-month data produced similar conclusions to the primary analyses (p=0.57 and 0.58, respectively).

There was no evidence of a difference between allocated groups in relation to the ward recommendation to family and friends or the CQUIN component of finding staff to talk to about worries/fears (p=0.44 and 0.92, respectively). In terms of the Hospital Survey of Patient Safety, there was also no evidence of a difference between allocated groups in the proportion of staff favourably grading their ward on perception of patient safety (p=0.22 and 0.87, respectively).

### Costs

Costs largely consisted of collection of patient data £24,463.80 (35%) and implementation of action plans £34,639.20 (50%). Implementation had a mean cost of £1018.80 (SD 1500.03) per ward. There was no information available to report whether actions plans related to the second cycle of action planning were implemented or not. It was assumed that those actions were partially implemented; therefore, implementations costs might be underestimated.

## Discussion

The RCT was successful in that all wards were retained throughout the study period and data were collected from 86% of patients approached. However, while patients were willing and able to provide feedback about safety, many ward teams found it difficult to meet to develop action plans and implement change.

The analyses reveal that there was no evidence of a difference in total HFC or the overall PMOS score between the allocated groups at 6 or 12 months. There was also no evidence of a difference in the new HFC score (those harms attributable to participating wards) between the allocated groups at 12 months. The difference between the two groups was 1.60% with an ES of 0.51 for this outcome. ESs were higher for differences in harm reduction between the intervention wards that complied with the intervention and matched controls. This suggests the possibility that for those wards that implement the intervention effectively, reductions in patient harm are feasible. It should be noted that the study was not powered for these analyses.

The main costs associated with the intervention arose from staff time (1) involved in the design and implementation of actions and (2) associated with patient data collection. Future models for the PRASE intervention might use volunteers to collect patient data that would reduce substantially the overall cost of the intervention. Indeed, this is an approach currently being piloted in three acute Trusts in the UK.

Possible explanations for the trial findings—measurement and fidelity—are discussed below.

### Measurement issues

Patient safety is universally acknowledged to be difficult to measure,^32^ particularly when the intervention is upstream with the potential to impact on a wide range of safety and quality outcomes. Our original primary outcome, the PMOS score, was also part of the intervention; therefore, we expected action plans would be focused on improving aspects of this and so we would be most likely to see an effect using this measure. The other primary outcome was the incidence of four patient safety harms on participating wards that was added to assess the impact on patient safety more generally. There were, however, some issues with this measure as high levels of HFC were routinely reported at baseline with a mean score of >90% and for new harms of >95%, meaning that achieving a substantial improvement within the trial was challenging. As with all routine NHS performance metrics, there is also the possibility of reporting bias; however, there was no reason that this would differ systematically between the control and intervention wards.

The intervention provides staff with feedback on the safety of the care environment, to which they are asked to respond by implementing local changes and making senior management aware of broader issues. This makes it difficult to predict in advance what changes a ward will choose to make and therefore what outcomes it might be appropriate to measure. One ward might focus on noise at night; another might focus on staff roles and responsibilities. It may not be possible to pick up improvement in these areas via the measurement of typical patient safety outcomes, for example, pressure ulcers, falls and venous thromboembolisms.

### Fidelity of intervention delivery

We collected information on the delivery of the intervention, following published guidance.^24^ The majority of wards met to consider feedback and many action planning groups included different types of staff. However, only three action planning teams included doctors and nine meetings were attended by only two people.

For each of the outcomes, and particularly new harms, the CACE analysis demonstrated larger differences between the allocated groups as adherence to the intervention improved. This suggests that when adherence is high and the ward is able to produce good quality action plans that are then implemented, safety improvements are more likely to follow. Unfortunately, within the current cohort of 17 intervention wards, only 4 were able to comply at this level, which illustrates the challenge of implementing changes to practice and clinical routines.

### What this study adds to the existing evidence base

A recent systematic review^33^ identified recommendations for which patient safety interventions were strongly encouraged for adoption based on current evidence. Interventions involving patient feedback or other patient involvement strategies did not appear on the list because there are too few rigorous studies of patient involvement in safety to provide a reliable evidence base. In recent years, a number of studies have investigated direct patient engagement in patient safety, for example, patient encouragement of hand hygiene practices.^6^ ^7^ Systematic reviews of patient engagement strategies have concluded that there is currently “insufficient high-quality evidence informing real-world implementation”.^17^ Moreover, there is currently no evidence available that addresses the specific question of whether patient feedback on patient safety has an impact on patient safety; therefore, direct comparison of our own work with other similar studies is not possible. However, current and previous studies on the feedback of patient-reported outcomes^34^ suggest that patients are willing and able to provide such feedback. What may be more difficult to address is the engagement of staff with this feedback and the use of this feedback by healthcare teams to improve services. We found that staff needed additional support to respond to patient feedback. This finding echoes that of a study of patient experience feedback^35^ in which feedback of patient experience to staff had little impact unless supported by ward meetings that were facilitated by research staff. In fact, in our trial, staff struggled to implement what seemed like relatively easy changes if they involved the need for support from other departments in the organisation, such as pharmacy or estates. These findings resonate strongly with recommendations of a recent report that evaluated the Health Foundation's Safer Clinical Systems initiative.^36^

### Strengths and limitations

This study represents the first RCT of the effectiveness of a patient feedback intervention to improve patient safety and was conducted across different wards from a range of hospitals. One of the key limitations of this trial was that we did not collect information about the impact of completing a questionnaire about safety on the patients themselves. It is possible that completing such a measure might lead to improvements in patients' knowledge and understanding of safety that could, in turn, lead to a more active role in monitoring safety but could also have the negative effect of inducing anxiety among patients who were previously unaware of the potential for things to go wrong. Our model for change focused on the feedback to staff and changes in their behaviour rather than patient-mediated change. Furthermore, it was not possible for the purpose of the trial to include patients who were unable to speak English or Mirpuri (a language spoken by the local Pakistani population). Thus, other patients were unable to take part. This limitation could be addressed by the translation of the materials into common languages spoken by the hospital population. However, for the purposes of the research this was not possible because we did not have the resources to employ multilingual research staff who would then be able to take consent from patients in the many different languages spoken across our hospital sites.

Also important to consider are the potential difficulties in the choice of outcome measures for interventions where flexibility in the changes implemented is actually encouraged. In other words, this was not an intervention that targeted falls and so where falls would be the obvious outcome measure. Ward teams involved in PRASE might choose to deliver one or more of a potentially finite number of changes, targeting many different patient safety issues, for example, speeding up the availability of medicines, reducing noise at night, removing clutter from corridors and making information about ward team roles available to patients. Measuring the impact of such changes without knowing in advance what they might be was not possible here. Agreement on how best to capture the impact of such upstream safety interventions is an important aim for researchers in this field.

## Conclusion

There is insufficient evidence from this cluster RCT to recommend the funding of the PRASE intervention. However, the trial demonstrates that patient reporting and feedback is feasible and acceptable to patients and that, where compliance with the intervention is high, there is potential for it to be effective in reducing patient harm.
