# Supplementary figures and images for: Can patient involvement improve patient safety? A cluster randomised control trial of the Patient Reporting and Action for a Safe Environment (PRASE) intervention
Source: BMJ Qual Saf. 2017 Feb 3;26(8):622–31. doi: 10.1136/bmjqs-2016-005570 (PMC5537521; doi:10.1136/bmjqs-2016-005570)

## Appendix 1: Outline logic model for the PRASE Intervention

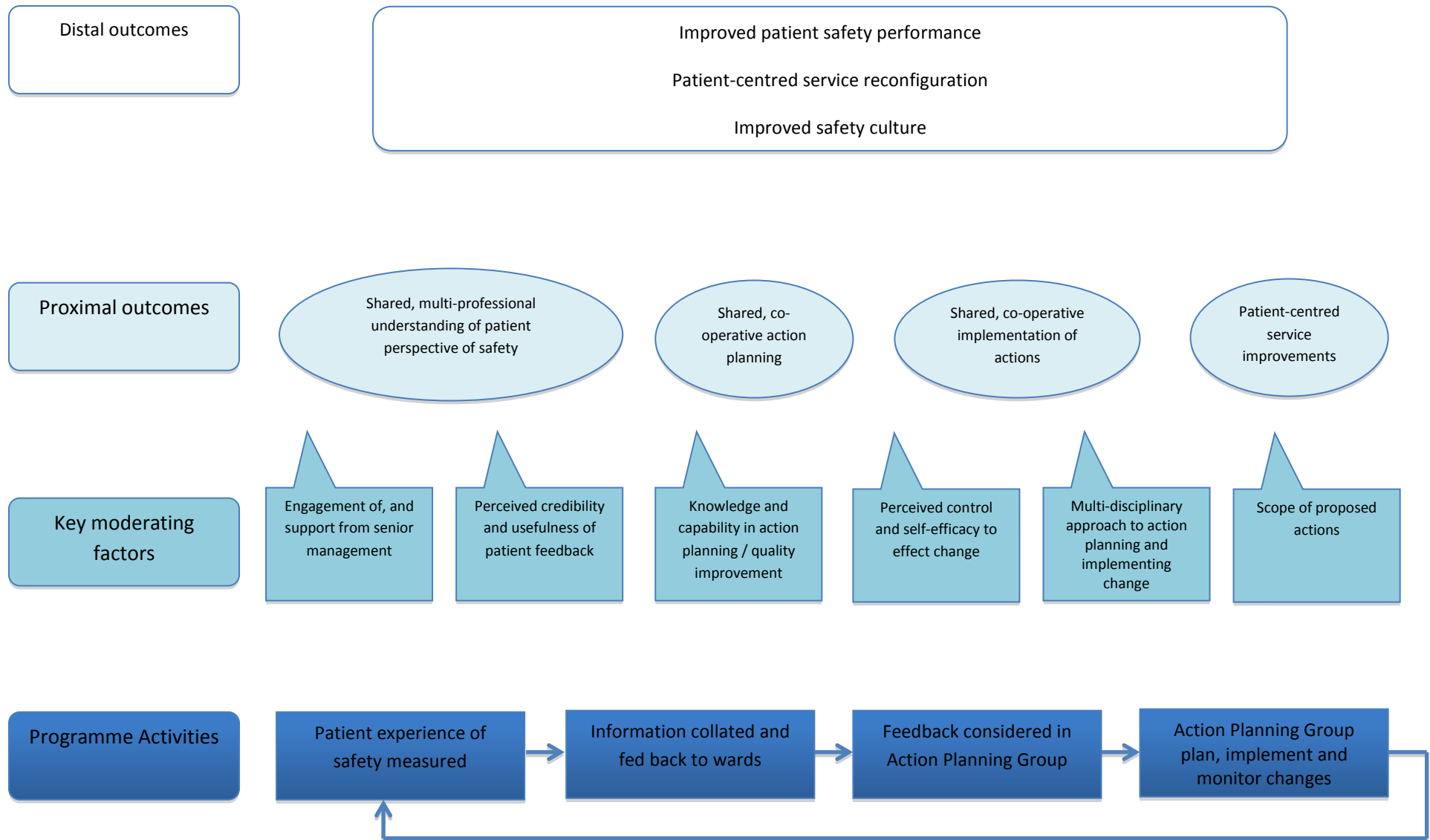

Supplement: supplementary appendix [file bmjqs-2016-005570supp001.pdf]
