# Supplementary material for: Can patient involvement improve patient safety? A cluster randomised control trial of the Patient Reporting and Action for a Safe Environment (PRASE) intervention
Source: BMJ Qual Saf. 2017 Feb 3;26(8):622–31. doi: 10.1136/bmjqs-2016-005570 (PMC5537521; doi:10.1136/bmjqs-2016-005570)
Supplement: supplementary appendix [file bmjqs-2016-005570supp002.pdf]

Appendix 2: Patient characteristics for PMOS respondents at each time point by allocation group

	Baseline		6 months		12 months	
	Control	*PRASE*	*Control*	*PRASE*	*Control*	*PRASE*
**N**	399	423	408	419	393	429
**Age**						
Mean (SD)	60.2 (18.2)	61.7 (17.6)	60.1 (18.4)	61.4 (17.9)	58.3 (18.9)	58.2 (18.6)
**Gender, n(%)**						
Female	189 (47.4)	199 (47.0)	181 (44.4)	185 (44.2)	200 (50.9)	201 (46.9)
Male	209 (52.4)	221 (52.2)	223 (54.7)	231 (55.1)	186 (47.3)	219 (51.1)
Missing	1 (0.3)	3 (0.7)	4 (1.0)	3 (0.7)	7 (1.8)	9 (2.1)
**Ethnicity, n(%)**						
Asian	9 (2.3)	11 (2.6)	11 (2.7)	9 (2.1)	10 (2.5)	7 (1.6)
Black	7 (1.8)	4 (0.9)	4 (1.0)	6 (1.4)	4 (1.0)	1 (0.2)
British/Irish	369 (92.5)	392 (92.7)	380 (93.1)	386 (92.1)	375 (95.4)	407 (94.9)
Other	10 (2.5)	10 (2.4)	9 (2.2)	13 (3.1)	3 (0.8)	10 (2.3)
Missing	4 (1.0)	6 (1.4)	4 (1.0)	5 (1.2)	1 (0.3)	4 (0.9)
